# Acute Toxicity and Prothrombotic Effects of Quantum Dots: Impact of Surface Charge

**DOI:** 10.1289/ehp.11566

**Published:** 2008-07-18

**Authors:** Jorina Geys, Abderrahim Nemmar, Erik Verbeken, Erik Smolders, Monica Ratoi, Marc F. Hoylaerts, Benoit Nemery, Peter H.M. Hoet

**Affiliations:** 1 Laboratory of Pneumology, Unit for Lung Toxicology, Katholieke Universiteit Leuven, Leuven, Belgium; 2 Faculty of Medicine and Health Sciences, Department of Physiology, United Arab Emirates University, Al-ain, United Arab Emirates; 3 Department of Pathology, Universitaire Ziekenhuizen Leuven, Leuven, Belgium; 4 Laboratory of Soil and Water Management, Katholieke Universiteit Leuven, Leuven, Belgium; 5 Department of Materials, University of Oxford, Oxford, United Kingdom; 6 Center for Molecular and Vascular Biology, Katholieke Universiteit Leuven, Leuven, Belgium

**Keywords:** blood cell analysis, coagulation, intravenous, *in vivo*, nanoparticles, platelet aggregation, quantum dots, surface charge, thrombosis, toxicity

## Abstract

**Background:**

Quantum dots (QDs) have numerous possible applications for *in vivo* imaging. However, toxicity data are scarce.

**Objectives:**

To determine the acute *in vivo* toxicity of QDs with carboxyl surface coating (carboxyl-QDs) and QDs with amine surface coating (amine-QDs), we investigated the inflammatory properties, tissue distribution, and prothrombotic effects after intravenous injection.

**Methods:**

We performed particle characterization by transmission electron microscopy and dynamic light scattering. Carboxyl-QDs and amine-QDs were intravenously injected in mice (1.44–3,600 pmol/mouse). At different time intervals, analyses included fluorescence microscopy, blood cell analysis, bronchoalveolar lavage, wet and dry organ weights, and cadmium concentration in various organs. We examined the prothrombotic effects *in vivo* by assessing the effect of pretreatment with the anticoagulant heparin and by measuring platelet activation (P-selectin), and *in vitro* by platelet aggregation in murine and human platelet-rich plasma exposed to QDs (1.44–1,620 pmol/mL).

**Results:**

At doses of 3,600 and 720 pmol/mouse, QDs caused marked vascular thrombosis in the pulmonary circulation, especially with carboxyl-QDs. We saw an effect of surface charge for all the parameters tested. QDs were mainly found in lung, liver, and blood. Thrombotic complications were abolished, and P-selectin was not affected by pretreatment of the animals with heparin. *In vitro*, carboxyl-QDs and amine-QDs enhanced adenosine-5′-diphosphate–induced platelet aggregation.

**Conclusion:**

At high doses, QDs caused pulmonary vascular thrombosis, most likely by activating the coagulation cascade via contact activation. Our study highlights the need for careful safety evaluation of QDs before their use in human applications. Furthermore, it is clear that surface charge is an important parameter in nanotoxicity.

Nanotechnology holds great promise in numerous fields, including medicine. However, concerns have been expressed about possible adverse effects of nanomaterials (Hoet et al. 2004; Oberdörster et al. 2005b; Xia et al. 2006). Although such concerns may have been exaggerated, they are not entirely without foundation (Maynard 2006), and it is fair to state that research and development of novel technologies, such as nanotechnology, often take place without much regard for potential harmful effects on the environment and/or human health (Maynard 2006). The medical applications of quantum dots (QDs) represent a case in point.

Nanosized QDs have specific properties—tunable emission wavelength, broadband absorption spectrum, and photostability—that make them ideal diagnostic probes for long-term *in vivo* and *in vitro* (multicolor) imaging (Ballou et al. 2004; Jaiswal et al. 2003; Larson et al. 2003; Medintz et al. 2005; Michalet et al. 2005; So et al. 2006) and site-specific targeting of drugs (Akerman et al. 2002; Gao et al. 2004; Stroh et al. 2005; Tada et al. 2007; Yu et al. 2007). The existing knowledge of the hazard of QDs is very incomplete, certainly compared with drugs or other chemical agents (Hardman 2006). Although the *in vitro* cytotoxicity of QDs has been studied (Derfus et al. 2004; Kirchner et al. 2005; Lovric et al. 2005a, 2005b; Shiohara et al. 2004), little or no attention has been paid to their potential *in vivo* toxicity (Ballou et al. 2004; Kim et al. 2004; Larson et al. 2003). Recently, some studies (Fischer et al. 2006; Gopee et al. 2007; Soo Choi et al. 2007; Yang et al. 2007) have investigated the biodistribution of QDs. However, most information on their *in vivo* toxicity—or the alleged lack of it—has been derived from studies that were not specifically designed to evaluate toxicity.

In the present study, we investigated the acute *in vivo* toxicity, including systemic and pulmonary inflammation, tissue distribution, and prothrombotic effects, of commercially available cadmium selenide/zinc sulfide (CdSe/ZnS) QDs. To assess the importance of surface charge, we studied QDs with different surface modifications [with carboxyl surface coating (carboxyl-QDs) or with amine surface coating (amine-QDs)]. We performed a detailed particle characterization, as has been recommended when studying the hazards of nanomaterials (Oberdörster et al. 2005a). Various doses—within the range of those used for *in vivo* applications (Ballou et al. 2004; Gao et al. 2004; Kim et al. 2004; So et al. 2006)—were intravenously (iv) administered to mice. In the first set of experiments, we administered relatively high doses (3,600, 720, or 144 pmol/mouse) and performed analyses after 1 hr. In the second set of experiments, lower doses (144, 14.4, or 1.44 pmol/mouse) were injected, and toxic effects were assessed over time (1, 4, or 24 hr). Because initial observations showed that injected QDs affected hemostasis, with vascular thrombi being produced in the lung circulation, we investigated the effect of an anticoagulant (heparin) on thrombosis *in vivo* and the effect of QDs on murine and human platelet aggregation *in vitro*. To our knowledge, this is the first comprehensive evaluation of the *in vivo* toxicity, prothrombotic effects, and biodistribution of injected QDs.

## Materials and Methods

### Particle characterization

Type I EviTags (Evident Technologies, New York, New York, USA) are core/shell QDs consisting of CdSe/ZnS. We used Catskill green, carboxyl or amine modified QDs (carboxyl-QDs and amine-QDs, respectively), purchased in a solution of approximately 9 nmol/mL QDs (or 0.25 mg/mL QDs). To ensure the quality of the QDs, we ran all experiments within 3 months after purchase and kept QDs in dark and refrigerated conditions. For the first set of experiments (144–3,600 pmol/mouse), this solution was concentrated 10-fold through evaporation of the solvent; for the second set of experiments (1.44–144 pmol/mouse), we used the original solution.

Particle size and distribution of the original solution and the concentrated solution were measured by dynamic light scattering (DLS), using a Malvern Zetasizer Nano-ZS (Malvern Instruments Ltd., Worcestershire, UK). To this end, 2 μL particles was dispersed in 800 μL water, Hanks’ Balanced Salt Solution (HBSS; Gibco, Merelbeke, Belgium), saline (0.9% NaCl), or medium Dulbecco’s Modified Eagle Medium (DMEM; Gibco). We performed three series of 15 measurements and calculated the average particle size. The zeta potential was calculated from electrophoretic mobility measurements in five series of 30 measurements (Malvern Zetasizer Nano-ZS; Malvern Instruments Ltd.). The isoelectric point (IEP) was determined in water by measuring the change in electrophoretic mobility as a function of pH: the solution to a pH of approximately 10 and acid was introduced—step by step—automatically by the Malvern Zetasizer. At each point, the zeta potential and size were measured. The IEP was calculated as the pH value when the charge around the particle becomes zero, that is, a net neutral surface charge. Sample preparation for transmission electron microscopy (TEM) consisted of placing a volume of 0.5 μL QDs on TEM copper grids (400 mesh) with a holey carbon film. The grids were then dried for 2–3 hr in a desiccator under vacuum. TEM pictures were taken using a JEOL 2010 microscope (JEOL Ltd., Hertfordshire, UK).

### Animals

Male Balb/c mice (20–23 g; Janvier Co., Uden, The Netherlands) received an iv injection in the tail vein (5 cm from distal end), while being held in a restraining device. A bolus injection of 40 μL sterile saline (0.9% NaCl) containing QDs or 40 μL saline (controls) was given using a plastic 0.3-mL syringe of U-100 insulin (BD Micro-fine; BD, Erembodegem, Belgium). We ran experiments on different days with the doses randomly organized, although we included at least one control each day. All experimental procedures were approved by the local ethical committee for animal experiments. Animals were treated humanely and with regard for alleviation of suffering.

### Experimental design

In the first set of experiments, 1 hr after injecting QDs (144–3,600 pmol/mouse), we removed the lung, heart, liver, kidneys, spleen, and brain for analysis by fluorescence microscopy. At the same time point, for QDs doses of 144–720 pmol/mouse, blood was collected, a bronchoalveolar lavage (BAL) was performed (right lung), and the weight (wet and after drying) of left lung, heart, liver, kidneys, spleen, and brain were recorded. Furthermore, in these organs and in the blood, we measured the Cd concentration to assess the tissue distribution.

In a separate experiment, mice were pre-treated with the anticoagulant heparin and a dose of QDs of 720 pmol/mouse was administered. One hour after injection, the blood was analyzed and the lung and liver were removed for evaluation by fluorescence microscopy. We performed flow cytometry to assess the expression of P-selectin on activated platelets.

In the second set of experiments, lower doses of QDs (1.44–144 pmol/mouse) were injected, and analyses were performed after 1, 4, and 24 hr: blood analysis, BAL, and wet and dry weight of left lung, liver, kidneys, and spleen. The Cd concentration was measured in lung, liver, and blood. We also performed *in vitro* platelet aggregation studies with human and murine platelet-rich plasma (PRP), as described below.

### Blood analysis

At 1, 4, or 24 hr after administration of QDs, the animals were anesthetized with 1.2 mg/kg pentobarbital (Nembutal; Ceva Sante Animale, Brussels, Belgium), and a blood sample was taken from the retroorbital plexus, using a heparinized capillary tube (Na-heparin minicaps; Hirschmann Laborgeräte, VWR, Haasrode, Belgium) filled with 4% sodium citrate (Sigma, Bornem, Belgium). The blood was collected in a tube containing heparin (BD microtainer with heparin) and analyzed for the number of erythrocytes, platelets, and total leukocytes, as well as neutrophils, lymphocytes, monocytes, eosinophils, and basophils (Cell-Dyn system 3500; Abbott Laboratories, Louvainla-Neuve, Belgium).

### Fluorescence microscopy

Animals were sacrificed 1 hr after injection of the QDs, by intraperitoneal injection of 90 mg/kg pentobarbital (Nembutal). A full-body perfusion via the left ventricle (with open vena cava) using a pressure of 1 m H_2_O was performed with 0.9% NaCl, followed by fixation with 4% formaldehyde (VWR, Haasrode, Belgium). We collected the organs and stored them separately in 4% formaldehyde for 24 hr and then in Sörensen buffer (Sigma). Pieces of each organ were embedded in paraffin wax and two sequential slices were prepared: one was stained with hematoxylin and eosin (H&E), and the other was not stained. Digital images were taken with an AxioCam HRc camera and AxioPlan microscope, both from Zeiss (Zaventem, Belgium).

### BAL and wet and dry organ weight

After sacrificing the animals, the chest was opened and the left lung was clamped, removed, and weighed (wet weight). After drying the lung at 70°C, we determined the dry weight. We also determined the wet and dry weights of other organs.

The right lung was lavaged three times with 0.4 mL sterile saline (0.9% NaCl). The recovered BAL fluid was pooled and centrifuged (2,000 × *g* for 10 min, 4°C). For differential cell counts, 250 μL of the resuspended cells (200,000 cells/mL) was spun (1,400 × *g* for 6 min; Shandon Cytospin 3; TechGen, Zellik, Belgium) onto microscope slides, air dried, and stained (Diff-Quik method). We then analyzed 3 × 100 cells for the presence of macrophages, eosinophils, neutrophils, or lymphocytes. We did not perform full-body perfusion in this part of the experiment.

### Assessing tissue distribution by measuring Cd concentration

After a full-body perfusion with sterile 0.9% NaCl, organs were collected. After drying, the organs were dissolved in 3 mL ultrapure nitric acid 60% (Sigma) in a water bath at 80°C, the Cd concentration was measured by inductively coupled plasma–optical emission spectrometry (ICP-OES; Optima 3300DV; Perkin Elmer, Zaventem, Belgium). The detection limit was 0.002 mg Cd/L, but only measurements > 0.006 mg Cd/L were considered positive. We calibrated the instrument with ICP Multi Element Standard Solution IV CertiPur (Sigma) containing 0.5 mg/L or 5 mg/L Cd. Blanks containing only nitric acid were measured as a negative control. The Cd concentration in the tissues is expressed on a wet weight basis, with mean ± SD weights of 1.275 ± 0.170 g for liver and 0.128 ± 0.032 g for lung, thus resulting in a detection limit of 0.014 μg Cd/g wet weight in liver and of 0.140 μg Cd/g wet weight in lung. In the high-dose experiments, 240 ± 48 μL blood was collected with a detection limit of 0.075 μg Cd/mL blood; in the low-dose experiments, 676 ± 98 μL blood was collected, resulting in 0.027 μg Cd/mL blood as the detection limit. The correlation between the amount of QDs and the concentration of Cd was obtained through a standard curve (1.08–90 pmol QDs).

### *Investigation of prothrombotic effects* in vivo

Hemostasis is subdivided into a primary and secondary cascade. The first refers to processes relating to platelet activation; the second, to processes of coagulation. Both processes are intertwined and occur to varying degrees in the arterial and venous circulation. We investigated whether the thrombosis was a result of activation of the coagulation cascade by administering QDs in mice pretreated with the anticoagulant heparin. Two minutes before iv injection of QDs (720 pmol/mouse; *n* = 3) or saline (*n* = 3), mice received an intraperitoneal injection of heparin (10 U, in 200 μL saline). One hour later, blood was sampled from the retroorbital plexus and cells were counted, as described above. After a full-body perfusion, we prepared the lung and liver of the mice for fluorescence microscopy, as described above. In addition, in the same animals, blood was collected to assess P-selectin as a measure of platelet activation. For this purpose, blood was collected from the retroorbital plexus, using a heparinized capillary tube filled with 4% sodium citrate, into an Eppendorf tube containing 20 μg/mL hirudin. This blood sample (100 μL) was fixed for 1 hr at 4°C in 900 μL diluted CellFix solution (BD). The fixed blood was centrifuged for 5 min at 2,000 rpm, the pellet was resuspended in 1 mL phosphate-buffered saline (PBS), and centrifuged for 5 min at 1,200 rpm. The pellet was kept at 4°C until analysis, within 5 hr.

For the analysis, the pellet was resuspended in 70 μL PBS with 20 μL blocking rat serum and 10 μL of an anti-CD62P antibody (phycoerythrin-labeled rat anti-mouse P-selectin; Emfret Analytics, Würzburg, Germany), and incubated for 20 min in the dark. Immediately after dilution to 1 mL with PBS, the samples were acquired on a FACSCaliber flow cytometer (BD) and the presence of P-selectin was investigated in the window of the platelets.

### In vitro *platelet aggregation tests*

We investigated whether the thrombosis resulted from activation of platelets by performing platelet aggregation tests. We prepared PRP by centrifuging citrated blood from untreated Balb/c mice and from six human healthy volunteers (men) who had not taken drugs, as previously described (Freson et al. 2001; Oury et al. 2003). The platelet count was adjusted to 0.25 × 10^6^ platelets/μL, via dilution in autologous plasma. We induced platelet aggregation, followed on a Chrono-log aggregometer (Kordia, Leiden, The Netherlands), with the agonist ADP (adenosine-5′-diphosphate), at 5 μM for murine platelet aggregation and 0.625, 1, or 1.5 μM for human platelet aggregation, depending on the response of the donor platelets to ADP. We added ADP 1 min after preincubation of the PRP with QDs and induction of stirring. We then added either carboxyl-QDs or amine-QDs to the plasma (1.44–1,620 pmol/mL). Tests were also performed without ADP.

### Statistical analysis

We analyzed differences between groups by one-way analysis of variance followed by Newman-Keuls test; the BAL samples were compared with a Student’s *t*-test (GraphPad Prism Package; GraphPad Software, San Diego, CA, USA). We considered *p*-values < 0.05 to be significant.

## Results

### Particle characterization

**Figure 1:**
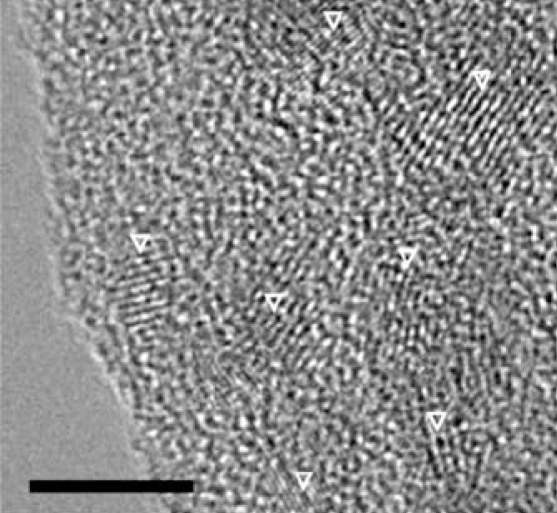
TEM of amine-QDs. The core/shell of the QDs can be seen as structured elements (arrowheads); the coating is spread as a layer over the whole grid. Bar = 5 nm.

**Figure 2:**
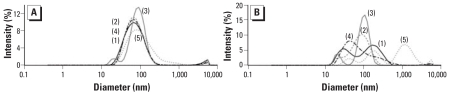
Size distribution by intensity, measured with DLS, of amine-QDs (*A*) and carboxyl-QDs (*B*) in water (1), HBSS (2), saline (3), DMEM (4), or water after 10-fold concentration through evaporation of the solvent (5).

We examined the QDs by TEM (Figure 1). The structured cores/shells of different QDs are apparent; the coating appears as an irregular mass in the background. With DLS and electrophoretic mobility, we measured the size and zeta potential in different vehicles, as listed in Table 1. According to the technical information and the material safety data sheets provided by the manufacturer, the QDs we used have a hydrodynamic diameter of approximately 40 nm. We found that the amine-QDs and carboxyl-QDs differed in diameter and poly-dispersity index, depending on the vehicle. Moreover, when we concentrated the QDs 10-fold, the particles tended to aggregate, and this was more pronounced with carboxyl-QDs than with amine-QDs, as illustrated in Figure 2. Both QDs have a negative zeta potential in all tested vehicles, but the carboxyl-QDs are 2-fold more negative than the amine-QDs (Table 1).

The core of the QDs consists of CdSe. Measurements in two different batches of particles revealed that approximately 27% of the QD mass consists of Cd (0.833 μg/mL QDs contains 0.23 mg/L Cd). The linear correlation (*R*^2^ = 0.9997) between Cd and QD concentration enables a quantification of the QDs in blood or tissues, down to 2.52 pmol.

### High doses of QDs: analysis 1 hr after injection

After iv injection of 3,600 pmol/mouse of carboxyl-QDs, three out of four mice died immediately; no deaths were recorded after injection of amine-QDs. After iv injection of 720 or 144 pmol/mouse, all mice showed signs of discomfort, such as labored breathing and piloerection, but no deaths occurred.

**Figure 3:**
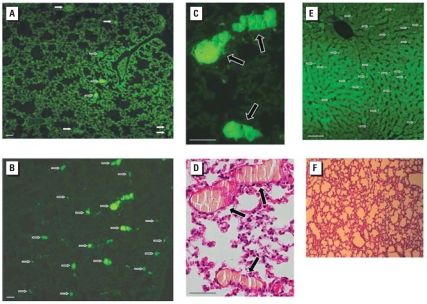
Fluorescence microscopy 1 hr after iv administration of QDs in mice. (*A* and *B*) Lung of mouse dosed with 3,600 pmol amine-QDs (*A*) or carboxyl-QDs (*B*). Arrows indicate fluorescent QDs in thrombi; note the difference in number of thrombi in (*A*) and (*B*). (*C* and *D*) Enlargement of thrombi (arrows) shown in (*B*). No platelets can be seen inside the thrombi in either nonstained sections (*C*) or H&E-stained sections (*D*); instead, fibrin fibers are present. (*E*) Liver of a mouse injected with 3,600 pmol amine-QDs; the arrows indicate QDs. (*F*) H&E staining of lung from a mouse pretreated with heparin and dosed with 720 pmol carboxyl-QDs shows complete absence of thrombi. Bars = 50 μm

Fluorescence microscopy revealed that particles were present in the lung and liver. Moreover, thrombi containing fluorescent QDs were apparent in the lung (Figure 3A,B). The number of thrombi was manifestly higher in animals injected with carboxyl-QDs than in those injected with amine-QDs. We found fewer thrombi at lower doses. H&E staining showed the presence of fibrin-rich thrombi in the lung circulation (Figure 3C,D). Fluorescent spots could also be found in the liver of mice treated with 3,600 pmol amine-QDs, but not at lower doses, and not with carboxyl-QDs (Figure 3E). We found no evidence of thrombi when the mice had been treated with heparin before injection of QDs (720 pmol; Figure 3F).

**Figure 4:**
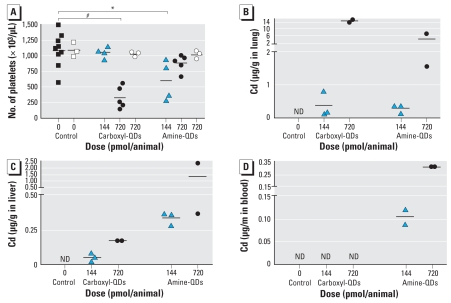
Effects and tissue distribution 1 hr after iv administration of carboxyl-QDs or amine-QDs (144 or 720 pmol/mouse). ND, below detection limit of 0.006 mg/L Cd. (*A*) Number of platelets in blood. Open squares and circles represent mice pretreated with heparin. (*B–D*) Tissue distribution based on the concentration of Cd in lung (*B*), liver (*C*), and blood (*D*). Horizontal bars represent the means. Blood analysis: control, *n* = 9; treatment, *n* = 4–5. Lung and liver analysis: control, *n* = 4; treatment, *n* = 2–3. ^*^p < 0.05.^#^*p* < 0.001.

One hour after dosing with 720 pmol/mouse of carboxyl-QDs, the platelet count was significantly reduced (Figure 4A). With amine-QDs, we saw only a slight decrease, reaching significance for 144 pmol/mouse. No changes occurred in total blood leukocyte numbers or in the percentages of neutrophils, lymphocytes, or monocytes (data not shown). In the BAL fluid, we found no influx of neutrophils or lymphocytes (data not shown). The wet and dry weights of the organs of mice injected with QDs were not different from those of controls (data not shown).

Pretreatment with heparin before injection of 720 pmol QDs abolished the drop in platelet count (Figure 4A). Flow cytometry showed that the platelet P-selectin levels in mice injected with QDs did not differ from those in control mice (data not shown); that is, we saw no evidence of *in vivo* platelet activation in the presence of heparin.

The Cd concentration was below the detection limit in all the collected organs of control animals. In animals treated with QDs, the Cd concentration in lung and liver was dose-dependently increased (Figure 4B,C). We found up to 15 μg Cd/g lung in animals that received 720 pmol of carboxyl-QDs, corresponding to 39% of the initial injected dose (Table 2). Animals dosed with 144 pmol carboxyl-QDs had a mean Cd content of 0.37 μg/g lung (a total of 0.048 μg Cd in lung), corresponding to 4% of the initial injected dose. The amount of Cd in the lungs of animals receiving amine-QDs was lower. In the liver, we found higher amounts of amine-QDs compared with carboxyl-QDs: 0.90 μg Cd/g liver and 0.33 μg Cd/g liver for 720 and 144 pmol amine-QDs, respectively, corresponding to 24% and 34% of the administered dose. In the blood, we found Cd only in mice that received amine-QDs (Figure 4D, Table 2). In the other organs tested, Cd concentrations were below the detection limit (data not shown).

### Low doses of QDs: analysis at different time points

To further assess toxicity over time (1, 4, and 24 hr), we selected doses that did not elicit noticeable acute symptoms in the mice (1.44–144 pmol/mouse). These doses are in the range of those used for *in vivo* applications (Kim et al. 2004; So et al. 2006).

**Figure 5:**
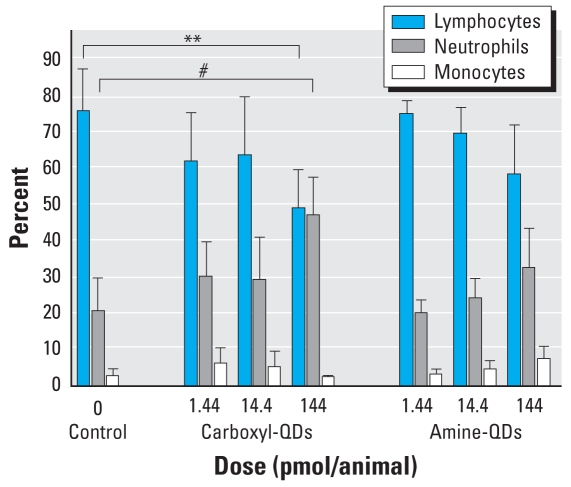
Leukocytes in blood (mean percentage ± SD) 4 hr after iv administration of carboxyl-QDs or amine-QDs (1.44, 14.4, or 144 pmol/mouse). Data represent mean ± SD. Control, *n* = 8; treatment, *n* = 6. **p < 0.01 for lymphocytes versus control. ^#^*p* < 0.001 for neutrophils versus control.

These doses did not decrease the number of platelets (data not shown). However, the percentage of neutrophils in blood significantly increased from 20% to 47% 4 hr after injecting 144 pmol of carboxyl-QDs; in parallel, the percentage of lymphocytes decreased from 75% to 48% (Figure 5). Absolute numbers of neutrophils significantly increased 1 hr after administering 144 pmol amine-QDs (2.77 ± 0.70 × 10^3^/μL vs. 1.54 ± 0.43 × 10^3^/μL neutrophils in controls, *p* < 0.05) and 4 hr after 144 pmol carboxyl-QDs (2.40 ± 0.98 × 10^3^/μL vs. 1.42 ± 0.28 × 10^3^/μL neutrophils in controls; *p* < 0.05). The number of monocytes was increased 4 hr after injecting 144 pmol carboxyl-QDs (0.46 ± 0.33 × 10^3^/μL vs. 0.17 ± 0.13 × 10^3^/μL monocytes in controls, *p* < 0.05) and 144 pmol amine-QDs (0.48 ± 0.18 × 10^3^/μL vs. 0.17 ± 0.13 × 10^3^/μL monocytes in controls; *p* < 0.05).

We found a significant increase in the number of macrophages in BAL fluid (28 ± 18 × 10^3^ macrophages in control) 4 hr after administering 1.44 pmol carboxyl-QDs (88 ± 46 × 10^3^ macrophages; *p* < 0.01) and 14.4 pmol amine-QDs (56 ± 22 × 10^3^ macrophages; *p* < 0.05). The wet and dry weights of the lung, liver, kidney, and spleen of mice injected with QDs did not differ from the controls (data not shown).

**Figure 6:**
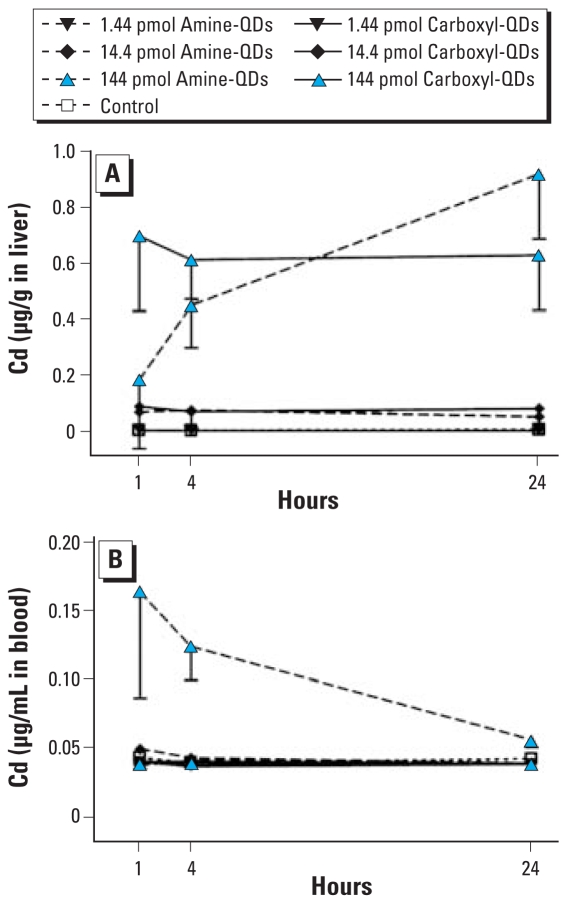
Tissue Cd distribution in liver (*A*) and blood (*B*) 1, 4, or 24 hr after iv administration of carboxyl-QDs or amine-QDs (1.44, 14.4, or 144 pmol/mouse). Control, *n* = 4; treatment, *n* = 3. Values below the detection limit (0.006 mg/L Cd) have been replaced with one-third of the detection limit.

Cd was detected in the liver of mice that received 144 or 14.4 pmol QDs (Figure 6A), whereas a dose of 1.44 pmol did not result in detectable amounts of Cd. For amine-QDs, the amount of Cd in the liver was higher after 24 hr (1.121 ± 0.251 total μg Cd, after injection of 144 pmol amine-QDs) than after 1 hr (0.237 ± 0.325 total μg Cd). For carboxyl-QDs, the amount of Cd in liver did not change over time (for 144 pmol carboxyl-QDs, 0.898 ± 0.291 total μg Cd after 24 hr vs. 0.904 ± 0.260 total μg Cd after 1 hr). In the blood, we found excess Cd only in mice that received amine-QDs, and the concentration decreased over time (Figure 6B). In the lung, we found no Cd (data not shown).

### In vitro *platelet aggregation tests*

**Figure 7:**
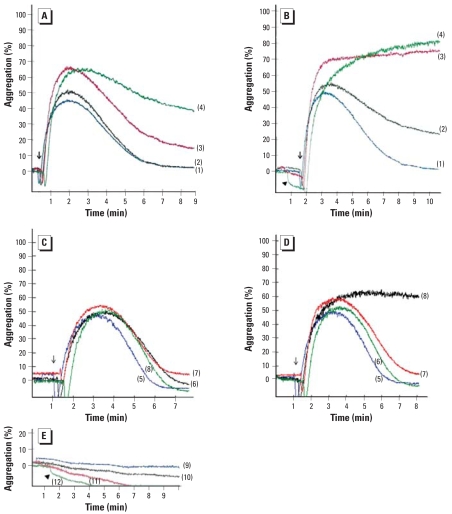
*In vitro* murine platelet aggregation in the absence or presence of ADP. (*A* and *B*) Murine PRP with agonist ADP; the tracings represent preincubation of PRP with different concentrations of amine-QDs (*A*) or carboxyl-QDs (*B*): 0 μg/mL (1), 180 pmol/mL (2), 540 pmol/mL (3), or 1,620 pmol/mL (4). Arrows indicate the time point of ADP addition. (*C* and *D*) Murine PRP with agonist ADP incubated with amine-QDs (*C*) or carboxyl-QDs (*D*) at 0 μg/mL (5), 1.44 pmol/mL (6), 14.4 pmol/mL (7), or 144 pmol/mL (8). (*E*) Murine PRP without ADP, incubated with amine-QDs at 540 pmol/mL (9) or 1,080 pmol/mL (10), or with carboxyl-QDs at 540 pmol/mL (11) or 1,620 pmol/mL (12). The arrowhead indicates the presence of platelet shape change.

To investigate whether human and murine platelets can be activated by QDs, we performed aggregation studies in PRP. The ADP-induced aggregation of murine PRP was dose-dependently enhanced upon preincubation for 1 min with amine-QD concentrations > 180 pmol/mL (Figure 7A,C). The effect of carboxyl-QDs on ADP-induced platelet aggregation was also dose dependent and even more pronounced. At 180 pmol/mL, platelet aggregation was elevated, leading to maximal platelet aggregation starting at 540 pmol/mL carboxyl-QDs (Figure 7B). Even a low dose of 144 pmol/mL carboxyl-QDs enhanced ADP-induced platelet aggregation; the lowest doses (14.4 and 1.44 pmol/mL) had no effect (Figure 7D). The particles themselves (amine-QDs or carboxyl-QDs) did not cause platelet aggregation in the absence of ADP. However, carboxyl-QDs induced a platelet shape change at a concentration of 1,620 pmol/mL (Figure 7E).

**Figure 8:**
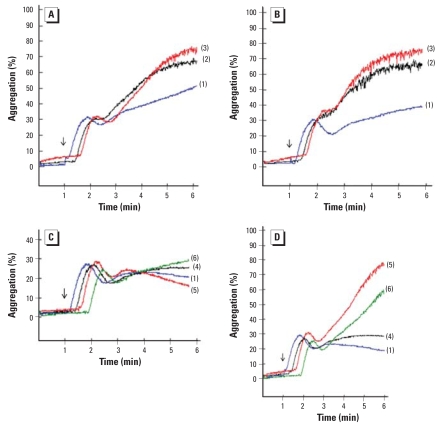
Effect of QDs on *in vitro* ADP-induced platelet aggregation of human PRP. (*A* and *B*) Human PRP from a single donor, incubated with amine-QDs (*A*) or carboxyl-QDs (*B*) at concentrations of 0 μg/mL (1), 360 pmol/mL (2), or 720 pmol/mL (3). (*C* and *D*) Human PRP from the same volunteer, incubated with amine-QDs (*C*) or carboxyl-QDs (*D*) at concentrations of 0 μg/mL (1), 1.44 pmol/mL (4), 14.4 pmol/mL (5), or 144 μg/mL (6). Arrows indicate the time point of ADP addition

Human PRP aggregation was more variably affected by QDs. Blood from four of six donors reacted on incubation with 720 pmol/mL amine-QDs with a mean 2.6-fold increase in maximal platelet aggregation amplitude, compared with control conditions without QDs; at 360 pmol/mL, only two of six donors responded, with a mean 1.7-fold increase. At lower concentrations, we noted no response. In the case of carboxyl-QDs, for one donor’s sample, the platelet aggregation was enhanced 3- or 4-fold at 144 or 14.4 pmol/mL, respectively. At 360 and 720 pmol/mL, four donors responded with a mean 2.6- and 3.7-fold increase, respectively. Figure 8A–D shows the platelet aggregation profile for one volunteer.

## Discussion

Despite industrial and medicinal promise of nanomaterials, concerns have been expressed about their possible adverse health effects (\Hoet et al. 2004; Oberdörster et al. 2005b; Xia et al. 2006). Because nanosized QDs are monodispersed and fluorescent, they can be used as good models for testing potential nanotoxicity. To study the influence of surface charge, we used commercially available type I EviTags, with either carboxyl or amine coatings; QDs of this type with neutral surface charge are not available. Because this is the first in-depth study specifically designed to test the *in vivo* toxicity of QDs, our purpose was to assess acute effects and to provide a dose–response relationship, together with an assessment of the tissue distribution, to identify the important target organs. Because vascular thrombi were produced in the lung, we investigated whether this thrombosis was due to either platelet activation or activation of coagulation. We examined platelet activation by assessing the effects of administering QDs on levels of P-selectin on circulating platelets (using flow cytometry) and by measuring platelet aggregation upon *in vitro* incubation of murine and human PRP with QDs. The role of coagulation was assessed by examining the effects of pretreatment with the anticoagulant heparin.

Experts have recommended characterizing essential physicochemical properties of nanoparticles whenever performing toxicity studies with nanomaterials (Oberdörster et al. 2005a). TEM is considered a gold standard for evaluating particle size distribution and shape (Oberdörster et al. 2005a). However, no satisfying TEM images could be obtained for the QDs used. The coating of the QDs was spread over the grid, covering the core/shell of the QDs, which is in line with findings of others (Fischer et al. 2006; Gopee et al. 2007; Soo Choi et al. 2007; Zhu et al. 2007). The separation of the coating from the core/shell of the QDs is thought to be due to the sample preparation process, including drying of the samples. As a consequence, no valuable data about aggregation and size could be obtained with this method. Nevertheless, sizes can be measured in solution using DLS. This method indicated that the amine-QDs were not aggregated, whereas the carboxyl-QDs were poly-dispersed in water (with the coexistence of two or three particle sizes) but almost mono-dispersed in the other tested vehicles. The zeta potential varied in the different vehicles: −14.2 mV for amine-QDs and −35.2 mV for carboxyl-QDs when dispersed in saline.

Because of their unique properties, QDs are considered ideal probes for *in vivo* imaging; low quantities (ranging from 300 pmol/g down to 0.01 pmol/g) are needed for *in vivo* applications (Ballou et al. 2004; Gao et al. 2004; Kim et al. 2004; Larson et al. 2003; So et al. 2006; Stroh et al. 2005; Tada et al. 2007; Yu et al. 2007). To establish realistic dose–response relationships, we tested concentrations from 3,600 pmol down to 1.44 pmol.

Three of four animals that received 3,600 pmol carboxyl-QDs died immediately upon injection because of massive thrombosis in the pulmonary vasculature. We also saw thrombi containing QDs in the lung of animals receiving 720 or 144 pmol carboxyl-QDs, and the number of thrombi was proportional to the dose administered. Carboxyl-QDs proved more potent in inducing pulmonary vascular thrombosis than did amine-QDs. A parallel drop in the circulating platelet count was induced, which at first suggested the occurrence of activation of circulating platelets, with aggregates being retained in the pulmonary microvasculature. However, although ADP-induced platelet aggregation was elevated by QDs *in vitro*, direct platelet activation by QDs does not explain the *in vivo* thrombotic complications. Indeed, the QDs themselves did not induce platelet aggregation, although the carboxyl-QDs induced a platelet shape change at the highest concentration tested, indicative of at least some platelet activation. For both surface properties, we observed a dose-dependent stimulation of the ADP-triggered platelet aggregation, and it was more pronounced for the carboxyl-QDs. The significance of these *in vitro* observations is unclear, because *in vivo* thrombotic complications were observed at QD plasma concentrations below those required for platelet activation stimulation.

This led us to hypothesize that the thrombotic events were caused not by platelet activation but by activation of the coagulation cascade, especially because massive fibrin fibers were present in the pulmonary thrombi. We tested this hypothesis by pretreating the mice with the anticoagulant heparin. With such pre-treatment, carboxyl-QDs no longer induced thrombosis in the lung; in addition, circulating platelet numbers were no longer decreased. Moreover, the platelet P-selectin levels indicated no evidence of *in vivo* platelet activation in the presence of heparin. These findings allow us to conclude that the negatively charged surface of carboxyl-QDs triggers coagulation, probably via contact activation, thus leading to fibrin formation and simultaneous platelet activation by thrombin, which is a potent agonist of platelet activation via PAR1 protease-activated receptors on platelets (Kataoka et al. 2003), which function is fulfilled by PAR4 receptors on mouse platelets. This explains the more pronounced effect of carboxyl-QDs compared with amine-QDs. In previous studies (Nemmar et al. 2002), we found that positively charged amine polystyrene nanoparticles enhanced the ADP-triggered platelet aggregation more strongly than did negatively charged carboxyl polystyrene nanoparticles. In contrast to the QDs used here, these polystyrene particles had no effect on coagulation tests (Nemmar et al. 2002). Those findings do not contradict our present data, where coagulation induction, rather than platelet activation, is triggered by the negatively charged QDs. Polystyrene beads have a different surface chemistry than do the presently studied QDs. In addition, the reported zeta potentials for the amine and carboxyl polystyrene beads were −44 mV and −41 mV (Nemmar et al. 2002), respectively, and hence not comparable with our QDs, with zeta potentials of −14.2 mV and −35.2 mV for amine-QDs and carboxyl-QDs, respectively.

The fluorescence microscopy observations are in agreement with the measurements of Cd in the organs. The relatively high Cd concentration in the QDs allowed concentrations down to 2.52 pmol to be detected by ICP-OES (assessed by a QD standard curve), resulting in a more powerful detection technique compared with fluorescence microscopy. In tissue distribution studies, QDs were found mainly in the lung, liver, and blood, with a difference between amine-QDs and carboxyl-QDs. Carboxyl-QDs were cleared from the blood 1 hr after iv injection. The absence of Cd in the kidney indicates that the QDs were not dissolved in the body during this time frame. Other biodistribution studies of QDs indicated liver, lung, kidney, and spleen as main sites of deposition (Fischer et al. 2006; Gopee et al. 2007; Soo Choi et al. 2007; Yang et al. 2007); the hydrodynamic diameter and the surface of the QDs are critical factors in blood half-life, tissue distribution pattern, and excretion (Fischer et al. 2006; Soo Choi et al. 2007). Surface coating, doses, and time windows examined in these studies differ significantly from our study.

The concentrations used in our toxicity study agree with the quantities needed for *in vivo* applications (Ballou et al. 2004; Gao et al. 2004; Kim et al. 2004; Larson et al. 2003; So et al. 2006; Stroh et al. 2005; Tada et al. 2007; Yu et al. 2007); however, linking our results to other studies is a challenge because the metrics defining dosage concentrations vary in the reported literature.

In conclusion, we conducted an acute *in vivo* toxicity study with commercially available carboxyl-QDs and amine-QDs using a large range of doses that encompass doses used for *in vivo* applications (3,600 pmol down to 1.44 pmol). We characterized the particles before administration and evaluated toxicity end points at different time points. At high doses, the QDs caused pulmonary vascular thrombosis, with carboxyl-QDs being more potent in inducing this effect than amine-QDs. Because fibrin fibers were present in the thrombi and because pretreatment with heparin abolished the thrombotic effects, we speculate that negatively charged QDs activate the coagulation cascade via contact activation. We saw an effect of surface charge in all the parameters tested. Lower doses of 14.4 pmol or 1.44 pmol did not elicit acute adverse effects. This is the first comprehensive evaluation of the hazard of injected QDs and emphasizes that surface charge is an important parameter in assessing nanotoxicity.

## Figures and Tables

**Table 1:** Physical characterization of the QDs.

Vehicle	Diameter (nm)^a^	Polydispersity index	Zeta potential (mV)	IEP
Amine-QDs				
DI Water	77.3	0.285	−32.2	pH 3.19
DI Water × 10^b^	137	0.331		
HBSS	80.5	0.249	−17.3	
Saline	93.3	0.328	−14.2	
DMEM	79.8	0.234	−12.0	
Carboxyl-QDs				
DI water	35.9, 186^c^	0.495	−57.7	pH 1.71
DI water × 10^b^	46.5, 188, 1,280^c^	0.946		
HBSS	84.5	0.291	−24.5	
Saline	104	0.352	−35.2	
DMEM	107	0.439	−24.3	

DI, deionized.

a Average diameter from intensity distribution, measured by DLS.

b The QDs were concentrated 10-fold through evaporation of the solvent.

c Samples were polydispersed, with the coexistence of two/three particle sizes.

**Table 2:** Cd measurement 1 hr after iv administration of carboxyl-QDs or amine-QDs (144 or 720 pmol/mouse).

	Lung		Liver		
Dose (pmol/mouse)	Total Cd (μg)^a^	Percent^b^	Total Cd (μg)^a^	Percent^b^	Blood (%)^b^
Carboxyl-QDs					
144	0.048 ± 0.042	4.4 ± 3.8	0.054 ± 0.028	4.9 ± 2.6	ND^c^
720	2.152 ± 0.386	39.1 ± 7.1	0.214 ± 0.017	3.9 ± 0.3	ND^c^
Amine-QDs					
144	0.043 ± 0.018	3.9 ± 1.7	0.377 ± 0.058	34.3 ± 5.2	2.3 ± 0.6
720	0.608 ± 0.500	11.0 ± 9.1	1.315 ± 1.227	23.9 ± 22.3	1.3 ± 0.3

ND, not detectable. Values are mean ± SD from two to three animals

a Total Cd in organ

b Percentage of the injected Cd dose

c < 0.006 mg/L Cd.
